# Acceptance and use of the distance education systems of Turkish medical educators during COVID-19 pandemic: an analysis of contextual factors with the UTAUT2

**DOI:** 10.1186/s12909-023-04024-7

**Published:** 2023-01-18

**Authors:** S. Koza Ciftci, Ramazan Gok, Engin Karadag

**Affiliations:** Faculty of Education, Akdeniz University, Antalya, 07070 Turkey

**Keywords:** COVID-19 pandemic, Distance education, UTAUT2, Medical educators

## Abstract

The aim of this study was to analyze the factors that has affected the use and approval of distance education systems during the COVID-19 pandemic in Turkey according to the extended Unified Theory of Acceptance and Use of Technology (UTAUT2). The study provided valuable insights on factors affecting the acceptance and use of distance education systems, which have become vital media of instruction since 2020. A total of 708 medical educators volunteered to participate in the study. The data were collected with a scale that was developed according to the UTAUT2 model. The scale consists of the variables of the UTAUT2 model as a ten-point Likert type questionnaire, including twenty-five items and seven dimensions: performance expectancy, effort expectancy, social influence, hedonic motivation, habits, facilitating conditions and behavioral intentions. The data were processed through correlation analysis, simple and multiple linear regression, and the structural equation model. The findings of the study indicated that performance expectancy, effort expectancy, social influence, hedonic motivation, habit, facilitating conditions and behavioral intentions all had positive effects on medical educators using distance education systems.

## Introduction

On December 31, 2019, the discovery of a new type of coronavirus (Covid 19) pneumonia-like infection in Wuhan, China, was reported to the World Health Organization (WHO). The virus was identified as causing serious health outcomes and even death [1]. In January of 2020, the Covid 19 infection evolved into a global pandemic affecting more than 160 countries, precipitating an unprecedented global problem. As a result, many countries temporarily closed all education institutions, including primary, secondary, and high schools, as well as universities, and decided to pursue instruction through distance education systems.

With regard to this pivot to online learning, A. Azoulay, the Director-General of UNESCO, remarked that, “We [have] entered a region without a map; that is, the borders have been crossed” [2]. On the other hand, some researchers have argued that educational systems were late to act on both a regional and global level. Similarly, a report by the OECD (2020) revealed that educators and administrators of educational institutions were lacking in terms of offering distance education, structuring online classes, and supporting students through the Covid 19 pandemic.

As with many other countries, Turkey resorted to distance education during the COVID-19 pandemic. On March 26^th^, 2020, Turkey’s Council of Higher Education (CoHE) announced that education would be delivered strictly via distance education, open education, and digital education systems for the spring academic semester [3]. It could be argued that universities, medical educators, and students were all caught unprepared for this sudden change. Therefore, examining the effectiveness of distance education during the pandemic was important to aid universities in planning their distance education policies for the future. In doing so, higher education institutions may be better prepared to develop a realistic perspective of their capacities and academic qualifications, as well as providing guidance for emergency remote teaching [4, 5] or distance education process, for planning alternative policies, and for improving the preparedness and competence of medical educators in terms of providing distance education [6–8]. With these issues in mind, this study aimed to reveal the factors affecting the acceptance and use of distance education systems by Turkish medical educators according to the Unified Theory of Acceptance and Use of Technology (UTAUT2) that was developed by Venkatesh et al. [9].

### Theoretical background and research hypotheses

The UTAUT2 theory was chosen because it is the most current and well-known technology acceptance theory, with superior explanatory capacity in contrast to other models [10]. In this sense, meta-analyses of the findings of studies carried out using the theory (e.g., [11, 12]) indicate that all the relationships between the structures of the model are important. UTAUT is a theory in which eight essential models and theories about the acceptance and use of a new technology have been experimentally combined by Venkatesh et al. [13]. The core constructs of the UTAUT theoretical framework include performance expectancy, effort expectancy, facilitating conditions, and social influence. However, Venkatesh et al. [9] developed an extended version of UTAUT in 2012, called UTAUT2, by adding three new constructs: hedonic motivation, habit, and price value.

The UTAUT2 was tested experimentally by Venkatesh et al. [9], and the direct effects explained 44% of the variance. When interaction terms were included, it explained 74% of the variance in behavioral intention. Likewise, in explaining technology use, UTAUT2's direct effects only model and moderated model explained 35% and 52% of the variance respectively, which indicates a significant increase in explained variance compared to the baseline/original UTAUT. These findings ensured that the basic dynamic structure of UTAUT2 comprises a useful tool for evaluating the adaptation levels of various technologies to estimate their prospective success rate for researchers. As such, many studies have utilized the UTAUT and UTAUT2 to test various technologies on different platforms, such as tablet computers [14–18], mobile devices/services [19–22], web sites [23], and Moodle or content management systems [24–27]. In addition, evaluations of new learning environments, such as mobile learning [28] and virtual learning environments [29, 30] using with UTAUT and UTAUT2 are also documented in the literature.

**Fig. 1:**
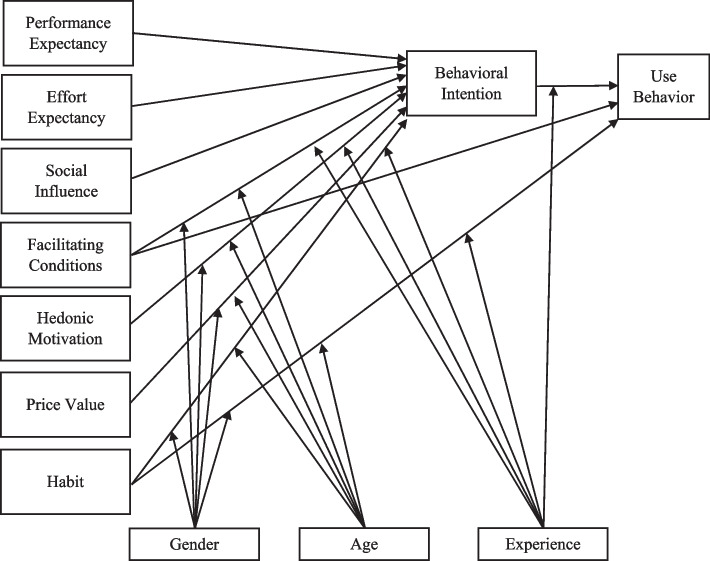
Model of UTAUT2 (Venkatesh et al. [9])

The UTAUT2 has seven basic constituents and three moderators. The basic dimensions of the model are performance expectancy, effort expectancy, social influence, facilitating conditions, hedonic motivation, habit, and price value, behavioral intention or use behavior. The moderators are gender, age, and experience, which have effects on the use behavior in the acceptance of technology [9] (see Figure 1).

Behavioral Intention (BI) is affected by the standard variables of the UTAUT2, performance expectancy, effort expectancy, social influence, hedonic motivation, habit, facilitating conditions and behavioral intention [9]. The UTAUT2 model assumes that if users accept that technology will improve their performance, they will employ it. Moreover, it has been reported that performance expectancy has positive or strongly positive effects on the behavioral intention [22, 31, 32]. Furthermore, Venkatesh et al. [13] argue that performance expectancy is the strongest predictor of behavioral intention, which has been also confirmed by a meta-analysis of 27 UTAUT studies [11]. Given these findings, the first hypothesis (H_1_) for the study was formulated as follows:

- **H**_**1**_ Performance expectancy has a positive impact on behavioral intention towards the use of distance education systems.

Another significant factor within the UTAUT2 is effort expectancy, which is defined as an internal element [33]. Because today's information technologies are user-friendly, and the technology literacy of the younger generation is high, effort expectancy is generally low. In this regard, Gupta et al. [32] and Venkatesh et al. [13] report positive effects of effort expectancy on behavioral intention. In this regard, effort expectancy has been found to be more effective for students who are experienced in e-learning than for users who have no experience with the technology in question [34]. As such, the second hypothesis (H2) was formulated as follows:

- **H**_**2**_ EE has a positive impact on behavioral intention towards the use of distance education systems.

Information technology and online social networks have changed social impact from physical to online and virtual environments. In this sense, it has been reported in the literature that social influence has positive or strongly positive effects on behavioral intention [35]. Social factors also have a strong but negative impact on the acceptance of e-learning systems [34], but the data for this study were collected from a sample of students. Therefore, the following hypothesis (H3) was formulated as follows:

- **H**_**3**_ SI has a positive impact on behavioral intention towards the use of distance education systems.

FC, which focus on control-related factors, is assumed in the original UTAUT model to affect use behavior directly [13]. Venkatesh et al. [13], on the other hand, argue that facilitating conditions have no significant effect on behavioral intention, and therefore, they used to facilitate conditions as a direct predictor of use behavior. In this regard, facilitating conditions are defined as the available sources and perception of support for individuals in carrying out a specific behavior [13]. Venkatesh et al. [13] conceptualized this factor using three variables in the current model: perceived behavioral control, compatibility and facilitating conditions. Hao et al. [36] confirmed the statistically significant effect of facilitating conditions on users’ behavioral intention. In addition, Venkatesh et al. [13] assumes that facilitating conditions may have statistically significant effects on the behavioral intention in terms of the acceptance of new technologies. Therefore, the following hypothesis (H4) was formulated for this study:

- **H**_**4**_ FC have a positive impact on behavioral intention towards the use of distance education systems.

HM, on the other hand, is defined as taking pleasure in using technology [9]. A perceived pleasure structure has been adopted in other models of acceptance of technology and is conceptualized as hedonic motivation. In this sense, if users enjoy themselves while applying technology, the chance of continuous use is much higher. Venkatesh et al. [9] further indicate that hedonic motivation has a statistically significant effect on the intent of users to employ technology; similarly, Brown and Venkatesh [37] argue that hedonic motivation is one of the basic predictors of behavioral intention to use technology. Therefore, the following hypothesis (H5) was formulated regarding hedonic motivation:

- **H**_**5**_ HM has a positive impact on behavioral intention towards the use of distance education systems.

Moreover, habit is defined as a tendency of individuals to carry out some acts automatically after learning them; in fact, habit is considered as a sensory construct [9]. Studies suggest that individuals who have used technology are easily affected by the technology at hand in the process of accepting it [38]. Venkatesh et al. [9] again argue that habit has statistically significant effects on users’ behavioral intention. Therefore, a hypothesis concerning habits (H6) was formulated as follows:

- **H**_**6**_ HTs have a positive impact on behavioral intention towards the use of distance education systems.

In addition, it has been revealed in various studies that behavioral intention affects the frequency of technology use. Therefore, the following hypothesis (H7) was formulated regarding behavioral intention:

- **H**_**7**_ Behavioral intention has a positive effect on usage behavior in distance education systems.

Both in the original UTAUT and in the expanded UTAUT2 models, it is assumed that gender has effects with regard to the relationship of performance expectancy, effort expectancy, and social influence toward behavioral intention [13]. In this respect, Ong and Lai [39] found that the scores of male participants were higher than those of women in all of the structures of the model. The effect of gender on some constructs of the model was supported in a study on e-learning environments in higher education [27]. Therefore, a hypothesis concerning gender (H8) was formulated as follows:

- **H**_**8**_ Gender has an effect on the relationships covered in the model.

Furthermore, in the UTAUT2 model, the ages of the participants are shown to have significant effects on some relationships [9]. Therefore, a hypothesis regarding age (H9) was formulated as follows:

- **H**_**9**_ Age has an effect on the relations covered in the model.

The price value component and experience moderator in the UTAUT2 were excluded from the research model in this study, since the distance education systems were provided by universities, and the education activities started at the same time as the COVID-19 pandemic.

## Methodology

### Design

This study aimed to explain the SEM, which was formed according to UTAUT2 and focused on medical educators’ acceptance and use of distance education systems through the structural equation mode and on whether the variables of performance expectancy, effort expectancy, social influence, facilitating conditions, hedonic motivation, and habit affect behavioral intention and usage behavior. At the first level of the model, the variables- performance expectancy, effort expectancy, social influence, hedonic motivation, and habit- were taken as the predictors, and behavioral intention as the result, whereas at the second level, the variables-behavioral intention, facilitating conditions, and habit- were taken as the predictors, usage behavior as the result, and age and gender as moderator variables [40].

### Participants

The population of the study consisted of 36,376 medical educators working at 113 universities in Turkey. *Email sampling* was used to choose the participants. The e-mail addresses were taken from the CoHE Academic [database] of the CoHE [41]. Using the technical background of UniAr (University Assessments & Research Laboratory), emails were sent to the faculty members between July 1st and July 30th, 2020 asking them to respond to the items in the data collection tool. A total of 708 medical educators (78 universities) responded by email (Table 1). Given that the reliability coefficient was 0.99 and the error margin was 0.05, the minimum sample to represent the 36,376 medical educators [42] was 661 [43]. Therefore, it was assumed that 708 participants represented the population appropriately.

**Table 1:** Demographic information of the participants

Variables		1	2	3	4	Total
Gender		Male	Female			
	*n*	388	320			708
	*%*	54.8	45.2			100
Academic Title		Professor	Associate professor	Assistant professor	Instructor	
	*n*	129	85	250	244	708
	*%*	18.2	12.0	35.3	34.5	100
Age		*M*: 43.08 *SD*: 9.6				

### The acceptance and use of the distance education systems scale

The data were collected through the scale that was designed for the purposes of the study. The items in the scale were developed based on the scale used in the study by Venkatesh et al. [9]. The scale used in the current study had two main differences from the scale developed by Venkatesh et al. [9]. First, the “experience” sub-scale in the original scale was not included, because before the COVID-19 pandemic, Turkish medical educators did not have experience with distance education. The second difference is related to the structure of the scale items; while the original scale items were developed based on “Mobile Internet,” the scale developed for this study was based on “Distance Education Systems.” The scale consists of 25 items and has a seven-factor structure. Higher scores indicate higher levels of acceptance and use of distance education systems. The questionnaire items were structured as a 10-point Likert scale ranging from 1 (totally disagree) to 10 (totally agree) [6]. To check the reliability of the scale, internal consistency was examined. Table 2 demonstrates the item numbers of the subscales and the corresponding Cronbach’s Alpha reliability coefficients.

**Table 2:** Cronbach’s Alpha reliability coefficients of the scales

Sub Scales	Number of Items	Cronbach’s Alpha
1-Performance Expectancy	4	.92
2-Effort Expectancy	4	.82
3-Social Influence	3	.89
4-Facilitating Conditions	4	.82
5-Hedonic Motivation	3	.95
6-Habit	4	.71
7-Behavioral Intention	3	.96

The following are example items from the scale:

• My interactions are clear and understandable with the distance education system.
• People who are significant to me think that the distance education system is useful.
• I find distance education useful in the teaching/learning processes.
• It has become ordinary for me to carry out the teaching/learning with distance education.
• Distance education has become a habit for me.
• I will always benefit from distance education in the teaching/learning processes.
• When I have difficulty in using distance education, I can get help from others.
• The use of distance education system is delightful.
• I should use the distance education system in future.
• I will always try to use distance education in my teaching profession.
• I am planning to employ the distance education systems frequently in future.

### Procedure

A research package containing questions items was created. The first three questions of the research package consisted of demographic questions: gender, age and academic status. The fourth question measured the “Behavior Frequency of Using Distance Education Systems” of the participants. This question used a ten-point Likert scale (1 = “Never or Almost Never” to 7 = “Pretty much”). The remaining 25 questions of the research package included The Acceptance and Use of the Distance Education Systems Scale. Afterwards, medical educators were contacted. First, the purpose of the study was explained to the medical educators, then informed consent forms were collected, and the participants were informed about the confidentiality of the data, as well as the voluntary basis and anonymity of their participation. Those who consented to participate were asked to respond to the questionnaire. It took approximately 10-15 minutes for the participants to complete the research package.

In this study, the relationships between The Acceptance and Use of the Distance Education Systems Scale scores of the participants were examined by correlation and regression analysis. Then, structural equation modeling (SEM) analysis was used to test the theoretical model via LISREL (ver. 8.51, Scientific Software International Inc, North Carolina, US.). The structural equation model was used to test the relationships between performance expectancy, effort expectancy, social influence, facilitating conditions, hedonic motivation, habit, and behavioral intention, which formed medical educators’ acceptance and use of distance education systems during the COVID-19 pandemic, according to theoretical model created by UTAUT2. The SEM, which was developed in accordance with UTAUT2, demonstrated the relationships between the determining factors: – namely, *performance expectancy, effort expectancy, social influence*, *hedonic motivation, habit* facilitating conditions - and behavioral intention and *use behavior*. While developing the model, it was assumed that performance expectancy, effort expectancy, social influence, hedonic motivation and habit variables would have an effect on behavioral intention; and, that behavioral intention and facilitating conditions would have an effect on the *use behavior*. Regarding the moderator variables, *age* was assumed to have an effect on performance expectancy, effort expectancy, social influence, facilitating conditions, hedonic motivation and habit; and *gender* was assumed to have an effect on effort expectancy, social influence, hedonic motivation and habit. The good fit indices used in the study were *X*^2^, *df*, Root Mean Square Error of Approximation (RMSEA), Goodness of Fit Index (GFI), Adjustment Goodness of Fit Index (AGFI), Normed Fit Index (NFI), Comparative Fit Index (CFI). In the data analysis performed in this study, significance was defined as *p*<.05.

### Findings

#### Normality assumptions and multicollinearity analysis

Before the data analysis, a complete behavioral intentions data set was created by determining the missing data by performing frequency analysis and assigning serial averages to the missing data. Subsequently, it was found that the data (*n* = 757) did not meet normality assumption (Kolmogorov-Smirnov *z* = 2.2.26-8.41, *p*<.01). Forty-nine replies, which were determined to be outliers using the z-score, were excluded from the analyzes and the analyzes were performed on 708 behavioral intentions. To check normality assumption again, Skewness (-.18 – .44) and Kurtosis [-.55 – .64] coefficients and the errors of these coefficients [Wed. Error: .08; Top. Error: .18] were calculated and since it was between -1.00 and +1.00, it was assumed to be within the normality limits of the skewness and kurtosis coefficients of the distribution. As a definitive indicator of normality, the Kolmogorov-Smirnov test (*p*=.36) was run and the results revealed normal distribution in terms of items and factor scores. Before the multivariate analysis, multicollinearity between the variables was checked by running Durbin-Watson (D-W) and VIF tests. Since the D-W value was 1.96 and the VIF values (2.29-3.41) were between 1<VIF<5, it was concluded that there was no multicollinearity. In addition, Q-Q graphs showed a normal distribution [44, 45].

#### Descriptive findings and correlation coefficients

Table 3 presents the correlation coefficients of the theoretical framework. As seen in Table 3, the highest coefficient was found for the dimension of “Facilitating Conditions”, (*M*=7.31, *SD*=1.77) and the lowest correlation was found for the dimension of “Hedonic Motivation” (*M*=4.79, *SD*=2.52). Correlation analysis on the variables in the theoretical model of the study showed that the results are statistically significant, and the correlation coefficients of these variables were found to be between .41 and .80.

**Table 3:** Results of the correlation analyses testing the theoretical model

Variables	*M*	*SD*	1	2	3	4	5	6	7
1-Performance Expectancy	5.13	2.37	-						
2-Effort Expectancy	7.10	1.88	.62^*^	-					
3-Social Influence	4.34	2.28	.77^*^	.51^*^	-				
4-Facilitating Conditions	7.31	1.77	.47^*^	.78^*^	.41^*^	-			
5-Hedonic Motivation	4.79	2.52	.80^*^	.60^*^	.73^*^	.46^*^	-		
6-Habit	5.69	1.91	.70^*^	.68^*^	.66^*^	.63^*^	.68^*^	-	
7-Behavioral Intention	5.05	2.72	.79^*^	.60^*^	.76^*^	.49^*^	.75^*^	.74^*^	-

^*^p <.01

#### Regression coefficients

Table 4 represents the unstandardized regression analysis results. The regression analyses, which were conducted for the variables in the theoretical model, were found to be statistically significant. The analyses primarily revealed that age predicts hedonic motivation, while behavioral intention was mainly predicted by performance expectancy and social influence. It was also found that habit had the highest prediction power regarding the use of technology.

**Table 4:** Results of the regression analyses testing the theoretical model

Variables	*B*	*β*	*S*_*e*_	*t*	*R*^*2*^
Age → Facilitating Conditions	-.02	-.12	.00	-3.41*	.01
Age → Hedonic Motivation	-.71	-.66	.02	-21.21*	.44
Age → Habit	-43	-.39	.02	-13.73*	.15
Performance Expectancy → Behavioral Intention	.97	.79	.02	34.49*	.62
Effort Expectancy → Behavioral Intention	.86	.60	.04	19.91*	.36
Social Influence → Behavioral Intention	.91	.76	.02	31.91*	.59
Facilitating Conditions → Behavioral Intention	.76	.49	.05	15.18*	.24
Hedonic Motivation → Behavioral Intention	.81	.75	.02	30.75*	.57
Habit → Behavioral Intention	1.05	.74	.03	29.19*	.54
Facilitating Conditions → Use Behavior	.61	.60	.03	20.33*	37
Habit → Use Behavior	.66	.70	.02	26.68*	.50
Behavioral Intention → Use Behavior	.40	.61	.01	20.90*	.38

^*^p<.01

Table 5 represents the results of the multiple-regression analysis conducted to calculate the explanatory power of the performance expectancy, effort expectancy and social influence subscales on the behavioral intention subscale. As seen from the table, the performance expectancy, effort expectancy, social influence, hedonic motivation, and habit subscales significantly explained 73% of the behavioral intention subscale in a positive direction [*R=*.85, *R*^*2*^=.73, *F*=389.78, *p*<.01].

**Table 5:** Results of the multiple regression analysis predicting the behavioral intention

*Behavioral Intention*	*B*	*S*_*e*_	*β*	*t*	*p*
(Constant)	-1.02	0.21		-4.80	0.00
1-Performance Expectancy	0.30	0.04	0.26	6.73	0.00
2-Effort Expectancy	0.05	0.04	0.04	1.27	0.21
3-Social Influence	0.32	0.04	0.27	7.99	0.00
4-Hedonic Motivation	0.18	0.04	0.16	4.65	0.00
5-Habit	0.35	0.05	0.24	7.62	0.00

*R=*.85, *R*^*2*^=.73, *F*=389.78, *p*<.01

Table 6 represents the results of the multiple-regression analysis conducted to calculate the explanatory power of the behavioral intention and facilitating conditions subscales on the use behavior subscale. As seen from the table, the behavioral intention and facilitating conditions subscales significantly explained 56% of the use behavior subscale in a positive direction [*R=*.74, *R*^*2*^=.56, *F*=298.97, *p*<.01].

**Table 6:** Results of the multiple regression analysis predicting use behavior

*Use Behavior*	*B*	*S*_*e*_	*β*	*t*	*p*
(Constant)	2.05	0.19		10.70	0.00
1-Behavioral Intention	0.13	0.02	0.19	5.23	0.00
2-Facilitating Conditions	0.26	0.03	0.25	7.84	0.00
3-Habit	0.37	0.04	0.40	9.61	0.00

*R=*.74, *R*^*2*^=.56, *F*=298.97, *p*<.01

#### Structural equation model

**Fig. 2:**
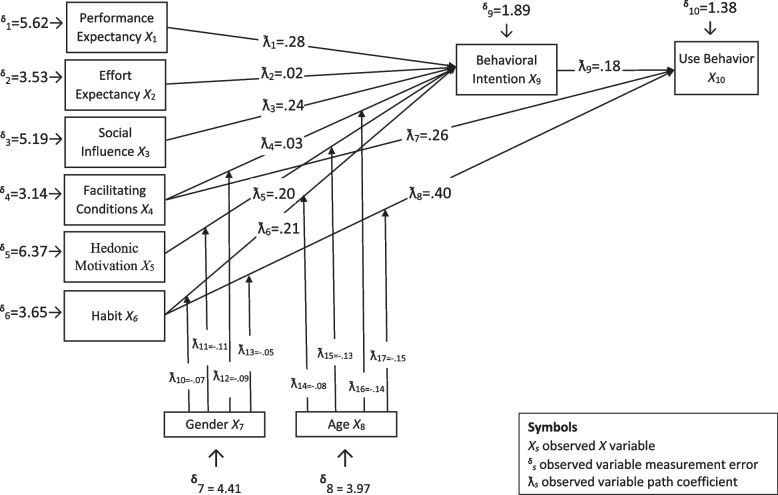
Structural equation diagram model of the acceptance and use of distance education systems: path coefficient

The results of the path analysis were given in Figure 2. In the model, it was found that behavioral intention was positively affected by performance expectancy, effort expectancy, social influence, facilitating conditions, hedonic motivation, and habit. Among these variables, performance expectancy was found to have the highest effect on behavioral intention. In addition, behavioral intention, facilitating conditions, and habit had positive effects on the use of technology. Moreover, the dimension of habits was found to have the highest effect on the use of technology. Therefore, it is possible to argue that all of the hypotheses were confirmed based on the values given in Figure 2.

#### Model prediction

Table 7 displays the goodness of fit indices of the variables contributing simultaneously to the model, which are the results of the path analysis performed for the theoretical model featuring the relationships among performance expectancy, effort expectancy, social influence, facilitating conditions, hedonic motivation, habit, behavioral intention, and use behavior regarding medical educators’ acceptance and use of distance education systems. It revealed that the models whose fit indexes were created corresponded to the data. This situation indicated that the model has had a significant predictive ability. However, the integration test between the models (delta-square) was found to be significant (*p<*.01). The chi-square (*X*^*2*^) value is related to the difference between the observed and expected values in the covariance matrices, and it is an undesirable behavioral intention result [46, 47]. If the model fit sample size increases, the value of the *X*^*2*^ statistic tends to exceed the fit limits. Therefore, the *p* value of the χ2 statistic is greatly affected by the sample size and results in the model being rejected unless there are very large samples [48]. Although the chi-square value was not expected to be significant in the SEM analysis, it can be said that the chi-square value was significant due to the sample size in the study [49–51].

**Table 7:** Fit indices of theoretical models

Model	Theoretic Model	Acceptable Values
*p*-value	<.001	>.05
*X*^2^/df	2.42	<3 [52]
RMSEA	.04	< .08 [53]
GFI	.98	≥.90 [52]
AGFI	.97	≥.85 [54]
NFI	.96	≥.90 [53]
CFI	.94	≥.90 [53]

#### Randomization test

The analyses in the study were carried out using data obtained from a non-random sample. Therefore, randomization tests were performed to support the generalizability of the findings beyond the study sample. In this regard, 5,000 bootstrap replicates were used to test the effects of the study variables. When the mean, standard errors, 95% confidence intervals, significance levels, and aspects of the relationships were examined, the bootstrapped samples showed that similar results were close to each other.

## Discussions, theoretical and practical implications

In the current study, the validity of the model proposed within the UTAUT2 was tested to reveal the factors affecting the acceptance and use of the distance education systems by Turkish medical educators during the COVID-19 pandemic. The effects of performance expectancy, effort expectancy, social influence, facilitating conditions, hedonic motivation, and habits, as well as gender and age as moderators, were examined. The findings indicated that all of the hypotheses were confirmed.

The confirmation of H_1_ suggested that performance expectancy has had a positive effect on behavioral intention. Similar findings have been reported in previous studies, particularly those focusing on the effects of performance expectancy on behavioral intention in e-learning environments [55–59]. Oye et al. [58], for example, found that the performance expectancy of medical educators had positive effects on their acceptance and use of information technologies in the workplace. In addition, similar results were reported in the studies in which Moodle [27] and personal computers [60] were used in relation to the effects of performance expectancy on behavioral intention. The positive effect of performance expectancy implied that use of distance education systems improves medical educators’ teaching performance. This positive effect could be interpreted to indicate that those medical educators with higher performance expectancy use distance education systems more frequently than those with lower performance expectancy, given that performance expectancy was found to have the highest effect on behavioral intention in this study, as also reported in previous studies [61, 62]. For this reason, it is safe to argue that medical educators have used distance education systems because of its positive effect on their teaching performance, rather than its being an easy way to deliver course content.

Moreover, the confirmation of H_2_ referred to the positive effect of effort expectancy on behavioral intention. This finding was consistent with the previous findings of studies which dealt with the effects of effort expectancy on behavioral intention [13, 18, 62–66]. In this sense, studies have shown that perceived ease of use, which creates the expectation of using CTI in teaching, has a positive effect on behavioral intention. For instance, Raman and Don [67] found a significantly positive effect of effort expectancy on pre-school teachers’ acceptance of learning management systems. Additionally, it could be stated that with the positive effect of effort expectancy, medical educators have had higher levels of intention to use distance education systems, as they found them easy to use [40]. This indicated assuming distance education systems as easy to use and user-friendly was an important factor. For this reason, it is important to focus on the difficulties experienced during the use of distance education in terms of software and hardware and on how to overcome these difficulties.

The confirmation of H_3_ denoted that social influence has had a positive effect on behavioral intention. This finding was consistent with previous findings [13, 18, 64–66]. There are also additional studies that have concluded that social influence affects behavioral intention, especially when technology use is mandatory [62, 63, 65]. Similarly, Fidani and Idrizi [68] confirmed a significant relationship between social influence and behavioral intention in terms of accepting a learning management system. Namely, the tendency of medical educators to use distance education systems due to the positive views of those who are important to them entails a positive effect of social influence. With this in mind, social influence may be used as a contributor to improve the intention of using distance education systems during the COVID-19 pandemic. Accordingly, if medical educators who adopted and used distance education systems interact with other medical educators in this regard, their use of distant education systems may increase substantially. For this reason, researchers should focus on ways to increase medical educators' acceptance and use of distance education.

The confirmation of both H_4_ and H_7_ referred to the fact that facilitating conditions and behavioral intentions have had positive effects on the use of distance education systems. This finding was consistent with previous findings [18, 61, 66]. The positive effect of facilitating conditions signaled the significance of the existence of institutional and technical infrastructure as contributing to the use of distance education systems. The positive effect of behavioral intention also referred to acceptance and to extension of a positive approach towards future use of distance education systems. For this reason, access to the resources required for the distance education should be facilitated, and information about the purposes for using distance education systems should be increased and supported in training programs. To support this, in-service training can be offered at universities at regular intervals; field experts may also provide consultancy to medical educators on an ongoing basis; and call centers may be established for access to problem-solving.

Moreover, the confirmation of H_5_ clearly indicated the positive effects of hedonic motivation on behavioral intention. This was consistent with previous findings (e.g., [9, 37, 55, 67, 69–72]). However, the finding contradicted that of Al-Gahtani [55]. The results showed that medical educators have had an acceptable level of internal motivation for using distance education systems. On the other hand, it would be beneficial for the developers of distance education systems to seek ways to reduce monotony. At the same time, however, it can be seen that behavioral intention was positively affected in particular when the use of technology was considered enjoyable.

The confirmation of H_6_ denoted a positive effect of habits on both behavioral intention and on the use of distance education systems. This finding was consistent with the previous findings. In this regard, a habit refers to the habitual or automatic behavior of individuals using technology. This may also be conceptualized as a perceptual construct that reflects the results of experiences [9]. Namely, over a long period of time, “constant use of technology becomes habitual, and this means that well-learned sequences of action can be activated by environmental clues and can then be repeated without conscious intent” [73]. As with the current study, previous research has suggested that habit is an important factor in predicting behavioral intention [9, 74, 75]. Davis and Venkatesh [76], for example, argued that habit is an alternative determinant of behavioral intention, as well as the use of technology. Moreover, it has also been found in numerous studies that habits directly affect behavioral intention [67, 69, 70, 72, 77, 78].

Regarding the effects of gender on the variables, it was found that facilitating conditions, hedonic motivation and habits had a much greater effect on the male participants’ behavioral intention in contrast to that of the female participants. The confirmation of H_8_ was also consistent with the previous findings [13, 79]. Therefore, it could be stated that it was easier for male participants to use the distance education systems than female participants, thus affecting their behavioral intention. As behavioral intention was a significant predictor of use behaviors, examining reasons why male users get adapted more easily could lead to higher levels of social impact, that is, the increase in the number of medical educators who intend to use distance education systems after a certain period of time.

Regarding the effects of age on the variables, it was observed that older participants had lower hedonic motivation and habits to use distance education systems so their behavioral intention was also lower respectively (confirmation of H_9_), however, age was a significant moderator variable on facilitating variables. This finding was consistent with previous findings [9]. This situation can be interpreted in the sense that decreases in hedonic motivation and habits are much more evident among older individuals, thus having a greater effect on their behavioral intention.

As a result, before the COVID-19 pandemic, online and distance education in medical education was heavily limited to live surgery observations. The pandemic has completely changed this situation, and especially preclinical medical education has moved online all over the world. The pandemic process was an extremely challenging process in terms of undergraduate medical education, requiring quick and sometimes instant decisions outside the defined processes existing in an uncertain environment. In this context, the pandemic process is a period when existing decision mechanisms should be strengthened, and it has increased our awareness of the importance of quality cycles in medical education and created an opportunity to accelerate studies in some areas- applications such as online measurement-evaluation. Again, despite the slowdown of the Pandemic and the transition to face-to-face education, many medical faculties have continued to utilize online learning tools as a support element, several of them even went further of this, for example, Akdeniz University Faculty of Medicine has started to implement the Flipped Learning Model since 2022.

### Limitations and directions for future research

The aim of this study was to analyze the factors that affected the use and acceptance of distance education systems during the COVID-19 pandemic in Turkish medical educators based on the extending the unified theory of acceptance and use of technology (UTAUT2). In this context, the limitations and suggestions for further research are as follows:

• If medical educators can be ensured that they will improve their teaching performance by utilizing distance education systems, their usage intention for these systems is likely to increase. Therefore, it may be important for researchers to conduct studies aimed at minimizing the difficulties in terms of technical infrastructure and software.
• Given the significance of social impact, the aims of medical educators to use distance education systems will be impacted by their colleagues. For this reason, an increase in the number of medical educators who use distance education systems effectively, as well as administrators with positive attitudes towards these systems, can change both the performance and the perceptions of users.
• In future studies, solutions to the problems faced by medical educators in using distance education systems may be offered. Information about these concerns and the variables that affect the use of acceptance and acceptance of distance education systems may lead to positive developments in this regard.
• In this study, the dimensions of acceptance and use of distance education systems by medical educators were examined only within the context of the COVID-19 pandemic. In future studies, analyses should be directed toward the use of distance education systems in an integrated manner throughout the formal education process.

The findings of the study suggested that stakeholders have also contributed to the acceptance and frequency of use of distance education systems by medical educators during the COVID-19 pandemic. It can also be added that these stakeholders seem to guide their use of distance education systems. In this respect, further studies may be conducted to develop more positive attitudes of stakeholders towards the use of distance education systems.

Furthermore, the analyses revealed that facilitating conditions explained 73% of the variance in behavioral intentions and behavioral intentions and facilitating conditions explained 56% in the use of the distance education systems. In this context, it should be considered that the unexplained variance may be caused by different variables. In the current study, the data were collected from only one country and evaluated concerning the compulsory distance education perspective during the COVID-19 pandemic. Thus, the generalizability of these findings was limited.

On the other hand, the approach of gathering the data through self-reports could cause the findings not to reflect the current status about the relationships between the variables due to participant subjectivity. In this regard, the most important methodological limitation of the current research is the *common method behavioral intentions* occurrence. The main reason for this limitation was that the data were collected from only a single source (medical educators). This situation may have caused the observed correlations to increase artificially. Although this limitation cannot be completely removed within the study, potential errors could be minimized. Accordingly, the necessary precautions were taken into consideration during the data collection process. First, the validity and reliability of the scale were tested, and secondly, the participants were told that the scales would be kept confidential and would not be shared under any circumstances. Additionally, the questionnaires have been designed in such a manner that the items related to the independent variables were positioned before the items related to the dependent variables.

## Data Availability

The data generated and/or analysed during the current study are not publicly available due to the ethics approval granted on the basis that only researchers involved in the study can access the de-identified data. The minimum retention period is five years from publication. Supporting documents are available upon request to the corresponding author.

## References

[1] Yuan J, Li M, Lu ZK (2020). Monitoring transmissibility and mortality of COVID-19 in Europe. Int J Infect Dis.

[2] Huang RH, Liu DJ, Tlili A, Yang JF, Wang HH, et al. Handbook on facilitating flexible learning: During educational disruption. Smart Learning Institute of Beijing Normal University; 2020. Retrieved from https://iite.unesco.org.

[3] The Council of Higher Education in Turkey. Üniversitelerde uygulanacak uzaktan eğitime ilişkin açıklama [Statement on distance education to be implemented in universities]. 2020. Retrieved from https://www.yok.gov.tr.

[4] Ferri F, Grifoni P, Guzzo T (2020). Online learning and emergency remote teaching: Opportunities and challenges in emergency situations. Societies.

[5] Trust T, Whalen J (2020). Should teachers be trained in emergency remote teaching? Lessons learned from the COVID-19 pandemic. J Technol Teacher Educ.

[6] Karadag E, Su A, Ergin-Kocaturk H (2021). Multi-level analyses of distance education capacity, faculty members’ adaptation, and indicators of student satisfaction in higher education during COVID-19 pandemic. Int J Educ Tech Higher Educ.

[7] Korkmaz G, Toraman Ç (2020). Are we ready for the post-COVID-19 educational practice? An investigation into what educators think as to online learning. Int J Technol Educ Sci.

[8] Polat M (2020). A rasch analysis of rater behavior in speaking assessment. Int Online J Educ Teach.

[9] Venkatesh V, Thong J, Xu X (2012). Consumer acceptance and use of information technology: Extending the unified theory of acceptance and use of technology. MIS Quarterly.

[10] Lee J, Rho MJ (2013). Perception of influencing factors on acceptance of mobile health monitoring service: a comparison between users and non-users. Healthc Inform Res.

[11] Dwivedi YK, Rana NP, Chen H, Williams MD. A Meta-analysis of the Unified Theory of Acceptance and Use of Technology (UTAUT). In Governance and sustainability in information systems. Managing the transfer and diffusion of IT. Edited by Nüttgens M, Gadatsch A, Kautz K, Schirmer I, Blinn N: Springer; 2011:155–70.

[12] Taiwo AA, Downe AG (2013). The theory of user acceptance and use of technology (UTAUT): A meta-analytic review of empirical findings. JATIT.

[13] Venkatesh V, Morris MG, Davis GB, Davis FD (2003). User acceptance of information technology: Toward a unified view. MIS Quarterly.

[14] Anderson J (2006). E Schwager, PH, Kerns RL: The drivers for acceptance of tablet PCs by faculty in a college of business. J Inform Syst Educ.

[15] El-Gayar O, Moran M, Hawkes M. Students’ acceptance of tablet PCs and implications for educational institutions. Educ Technol Society. 2011;14:58–70.

[16] Garfield MJ (2005). Acceptance of ubiquitous computing. Inform Syst Manag.

[17] Ifenthaler D, Schweinbenz V (2013). The acceptance of Tablet-PCs in classroom instruction: The teachers’ perspectives. Computers in Human Behavior.

[18] Moran M, Hawkes M, El Gayar O (2010). Tablet personal computer integration in higher education: Applying the unified theory of acceptance and use technology model to understand supporting factors. J Educ Comput Res.

[19] Carlsson C, Carlsson J, Hyvonen K, Puhakainen J, Walden P. Adoption of mobile devices/services-searching for answers with the UTAUT. In Proceedings of the 39th Annual Hawaii International Conference on System Sciences. 2006; Kauai.

[20] Sharif A, Afshan S, Qureshi MA (2019). Acceptance of learning management system in university students: an integrating framework of modified UTAUT2 and TTF theories. Int J Technol Enhanced Learning.

[21] Shin DH, Shin YJ, Choo H, Beom K (2011). Smartphones as smart pedagogical tools: Implications for smartphones as u-learning devices. Computers in Human Behavior.

[22] Zhou T, Lu Y, Wang B (2010). Integrating TTF and UTAUT to explain mobile banking user adoption. Computers in Human Behavior.

[23] Van Schaik P (2009). Unified theory of acceptance and use for websites used by students in higher education. J Educ Comput Res.

[24] Hsu HH (2012). The acceptance of Moodle: An empirical study based on UTAUT. Creative Education.

[25] Tseng TH, Lin S, Wang YS, Liu HX (2022). Investigating teachers’ adoption of MOOCs: the perspective of UTAUT2. Interact Learn Environ.

[26] Marchewka JT, Liu C, Kostiwa K (2007). An application of the UTAUT model for understanding student perceptions using course management software. Communications of the IIMA.

[27] Šumak B, Polancic G, Hericko M. An empirical study of virtual learning environment adoption using UTAUT. In International Conference on Mobile, Hybrid, and On-Line Learning. Edited by Adam M. Gadomski, Bernd Krämer, Cynthia Y. Lester, Manuela Popescu: Conference Publishing Services; 2010:17–22.

[28] Williams PW. Assessing mobile learning effectiveness and acceptance. PhD thesis. The George Washington University; 2009.

[29] Arain AA, Hussain Z, Rizvi WH, Vighio MS (2019). Extending UTAUT2 toward acceptance of mobile learning in the context of higher education. UAIS.

[30] Van Raaij EM, Schepers JJ (2008). The acceptance and use of a virtual learning environment in China. Comput Educ.

[31] Casey T, Wilson-Evered E (2012). Predicting uptake of technology innovations in online family dispute resolution services: An application and extension of the UTAUT. Computers in Human Behavior.

[32] Gupta B, Dasgupta S, Gupta A (2008). Adoption of ICT in a government organization in a developing country: An empirical study. J Strategic Inform Syst.

[33] Yoo SJ, Han SH, Huang W (2012). The roles of intrinsic motivators and extrinsic motivators in promoting e-learning in the workplace: A case from South Korea. Computers in Human Behavior.

[34] Cheng Y, Yu T, Huang C, Yu C, Yu C (2011). The comparison of three major occupations for user acceptance of information technology: Applying the UTAUT model. I-Business.

[35] Im I, Hong S, Kang MS (2011). An international comparison of technology adoption: Testing the UTAUT model. Inform Manag.

[36] Hao S, Dennen VP, Mei L (2017). Influential factors for mobile learning acceptance among Chinese users. Educ Technol Res Dev.

[37] Brown SA, Venkatesh V (2005). A model of adoption of technology in the household: A baseline model test and extension incorporating household life cycle. MIS Quarterly.

[38] Crabbe M, Standing C, Standing S, Karjaluoto H (2009). An adoption model for mobile banking in Ghana. IJMC.

[39] Ong CS, Lai JY (2006). Gender differences in perceptions and relationships among dominants of e-learning acceptance. Computers in Human Behavior.

[40] Tosuntas SB, Karadag E, Orhan S (2015). The factors affecting acceptance and use of interactive whiteboard within the scope of FATIH project: A structural equation model based on the unified theory of acceptance and use of technology. Comput Educ.

[41] YOK Academic Search Database. 2020. Retrieved from: https://akademik.yok.gov.tr.

[42] The Council of Higher Education in Turkey. Yükseköğretim istatistikleri [Higher education statistics]. 2020. Retrieved from https://istatistik.yok.gov.tr/.

[43] Hamburg M (1985). Basic Statistics: A Modern Approach.

[44] Özdamar K. Paket programlar ile istatistiksel Veri Analizi-1: SPSS-MINITAB. Kaan Kitabevi; 2009.

[45] Sipahi B, Yurtkoru S, Çinko M (2006). Sosyal Bilimlerde SPSS’le Veri Analizi [Data Analysis Using SPSS In Social Sciences].

[46] Munro BH. Statistical methods for health care research (Vol. 1). Lippincott Williams & Wilkins; 2005.

[47] Schumacker RE, Lomax RG. A Beginner’s Guide to Structural Equation Modeling. New York: Taylor & Francis Group; 2010. p. 85–90.

[48] Waltz CF, Strcikland OL, Lenz ER (2010). Measurement in Nursing and Health Research.

[49] Jöreskog KG, Sörbom D, Du Toit S, Du Toit M (2001). LISREL 8: New Statistical Features.

[50] Gerbing DW, Anderson JC (1984). On the meaning of within-factor correlated measurement errors. Journal of Consumer Research.

[51] Schumacker RE, Lomax RG. A Beginner’s Guide to Structural Equation Modeling. New Jersey: Taylor & Francis; 2004. p. 1–8.

[52] Kline RB (2005). Principles and Practice of Structural Equation Modeling.

[53] Hu LT, Bentler PM (1999). Cutoff criteria for fit indexes in covariance structure analysis: Conventional criteria versus new alternatives. SEM: A Multidiscip J.

[54] Cole DA (1987). Utility of confirmatory factor analysis in test validation research. J Consult Clin Psychol.

[55] Al-Gahtani SS (2016). Empirical investigation of e-learning acceptance and assimilation: A structural equation model. Appl Comput Inform.

[56] Chu TH, Chen YY (2016). With good we become good: Understanding e-learning adoption by theory of planned behavior and group influences. Comput Educ.

[57] Chiu CM, Wang ET (2008). Understanding Web-based learning continuance intention: The role of subjective task value. Inform Manag.

[58] Oye ND, Iahad NA, Rahim NA (2014). The history of UTAUT model and its impact on ICT acceptance and usage by academicians. Educ Inform Technol.

[59] Merhi MI (2015). Factors influencing higher education students to adopt podcast: an empirical study. Comput Educ.

[60] El-Gayar OF, Moran M. College students’ acceptance of Tablet PCs: An application of the UTAUT Model. In: 36th Annual Meeting of the Decision Sciences Institute (DSI). 2006.

[61] Meng H, Wang T. Acceptance of IWBs instruction and concomitant behavior through self-regulation learning. GSTF Journal on Computing. 2012;1:1–5.

[62] Wong K, Russo S, McDowall J (2013). Understanding early childhood student teachers’ acceptance and use of interactive whiteboard. CWIS.

[63] Wong K, Teo T, Goh PSC (2015). Understanding the intention to use interactive whiteboards: model development and testing. Interact Learn Environ.

[64] Teo T. Modeling the determinants of pre-service teachers’ perceived usefulness of e-learning. CWIS. 2011;28:124–40.

[65] Venkatesh V, Davis FD (2000). A theoretical extension of technology acceptance model: Four longitudinal field studies. Manag Sci.

[66] Wang Y, Shih Y (2009). Why do people use information kiosks? A validation of the unified theory of acceptance and use of technology. Gov Inf Q.

[67] Raman A, Don Y (2013). Preservice teachers’ acceptance of learning management software: An Application of the UTAUT2 Model. Int Educ Stud.

[68] Fidani A, Idrizi F. Investigating students’ acceptance of a learning management system in university education: a SEM approach. ICT Innovations Proceedings; 2012;2:311–20.

[69] Agudo-Peregrina AF, Hernandez-García A, Pascual-Miguel FJ (2013). Behavioral intention use behavior and the acceptance of electronic learning systems: Differences between higher education and lifelong learning. Comput Human Behav.

[70] Alalwan AA, Dwivedi YK, Rana NP, Lal B, Williams MD (2015). Consumer adoption of Internet banking in Jordan: Examining the role of hedonic motivation, habit, self-efficacy and trust. J Financ Serv Mark.

[71] Chen Y, Lin Y, Yeh R, Lou S (2013). Examining factors affecting college students’ intention to use web-based instruction systems: Towards an integrated model. TOJET.

[72] Lewis CC, Fretwell C, Ryan J, Parham JB (2013). Faculty use of established and emerging technologies in higher education: A unified theory of acceptance and use of technology perspective. Int J High Educ.

[73] Bandyopadhyay K, Fraccastoro KA (2007). The effect of culture on user acceptance of information technology. Commun Assoc Inf Syst.

[74] Kim SS, Malhotra NK, Narasimhan S (2005). Research note—two competing perspectives on automatic use: A theoretical and empirical comparison. Info Syst Res.

[75] Lim H, Lee SG, Nam K (2007). Validating E-learning factors affecting training effectiveness. Int J Info Manag.

[76] Davis FD, Venkatesh V (2004). Toward pre-prototype user acceptance testing of new information systems: Implications for software project management. Eng Manag IEEE Trans.

[77] Arenas Gaitán J, Peral Peral B, Ramón Jerónimo M (2015). Elderly and internet banking: An application of UTAUT2. JIBC.

[78] Escobar-Rodríguez T, Carvajal-Trujillo E (2013). Online drivers of consumer purchase of website airline tickets. JATM.

[79] Orji RO (2010). Impact of gender and nationality on acceptance of a digital library: an empirical validation of nationality based UTAUT using SEM. J Emerg Trends Comput Inf Sci.

